# Impact of NA‐1 on Pericyte‐Driven Vasoconstriction and Its Role in No‐Reflow During Cerebral Ischemia–Reperfusion

**DOI:** 10.1111/cns.70409

**Published:** 2025-05-25

**Authors:** Xinxuan Yang, Jiahui Zhao, Hao Tian, Ximing Nie, Lina Zheng, Xin Liu, Zheng Z. Wei, Yuchuan Ding, Liping Liu

**Affiliations:** ^1^ Department of Neurology, Beijing Tiantan Hospital Capital Medical University Beijing China; ^2^ China National Clinical Research Center for Neurological Diseases Beijing China; ^3^ Department of Neurology, Beijing Friendship Hospital Capital Medical University Beijing China; ^4^ Department of Neurosurgery Wayne State University School of Medicine Detroit Michigan USA

**Keywords:** cerebral reperfusion, NA‐1, no‐reflow phenomenon, pericytes

## Abstract

**Background:**

The no‐reflow phenomenon in the ischemic brain following arterial recanalization leads to poor prognosis. Previous studies suggest that the clinically proven NA‐1 drug, with disruption of PSD95 in the neuronal terminals alongside the cerebral microvasculature, may inhibit pericytic contraction of microvessels by reducing endothelin‐1 secretion.

**Methods:**

A 1.5‐h tMCAO model using Balb/c mice was employed. In vivo two‐photon imaging and immunofluorescence staining were employed to assess the constriction effect of pericytes on capillaries. The impact of NR2B9c/NA‐1 administration (intravenously infused at a dose of 10 μmol/kg) on pericyte constriction was evaluated through immunofluorescent staining of brain sections. Additionally, The effect of NA‐1 on cerebral perfusion was assessed using laser speckle blood flow monitoring, whole brain slice perfusion, and high‐magnification capillary imaging. Enzyme‐linked immunosorbent Assay (ELISA) was conducted to determine changes in quantitative ONOO^−^ and endothelin‐1 (ET‐1) content after tat‐NA‐1 administration. Lastly, microtubule‐associated protein 2 (MAP2) staining and behavioral scores were used to evaluate the effects of NA‐1 on infarct size and behavioral deficits.

**Results:**

In vivo two‐photon imaging and immunofluorescence staining revealed that pericyte constriction following ischemia and recanalization resulted in a decreased diameter of capillaries, particularly at the soma and adjacent areas. Notably, capillary obstruction was localized near pericytes. After administration of NA‐1, the immunofluorescence staining section showed that the diameter of capillaries at pericytes' soma and in the adjacent parts increased. The ELISA results indicated a reduction in ONOO^−^ and ET‐1 levels. Additionally, MAP2 staining revealed a decrease in infarct size, while behavioral scores showed improvements in deficits. This effect of NA‐1 was counteracted notably when added ET‐1.

**Conclusion:**

NA‐1 inhibits pericyte constriction following ischemia–reperfusion by reducing ET‐1 levels. It improves capillary no‐reflow in mice, enhances cerebral perfusion, decreases infarct size, and mitigates behavioral deficits.

## Graphical abstract

In this work, we provided cues that administering NA‐1 treatment reduced endothelin‐1 levels and inhibited pericyte‐mediated vasoconstriction, thereby improving cerebral perfusion and the no‐reflow phenomenon after ischemia‐recanalization, reducing infarct size and behavioral deficits, and improving prognosis.

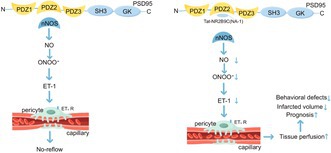

**Abbreviations**
CCA: common carotid artery
ECA: external carotid artery
ELISA: Enzyme‐Linked Immunosorbent Assay
ET‐1: endothelin‐1
ICA: internal carotid artery
LSCI: laser speckle contrast imaging
MAP 2: microtubule‐associated protein
MCAO: middle cerebral artery occlusion
mNSS: modified neurological severity score
NO: nitric oxide
ONOO^−^
: peroxynitrite
PSD95: Postsynaptic Density Protein 95
ROS: reactive oxygen species
tMCAO: transient middle cerebral artery occlusion

## 1 Introduction

Stroke is one of the leading causes of death and disability worldwide [1, 2]. Vascular recanalization is crucial for restoring local blood flow and offers hope to patients suffering from acute ischemic stroke [3]. However, achieving consistent and sustainable tissue perfusion after reopening the blood vessels is often difficult, which presents a significant challenge. This is particularly concerning because the failure to achieve effective reperfusion, known as the “no‐reflow” phenomenon, is closely linked to disturbances in the microvasculature, which are key pathophysiological changes [4].

Understanding the underlying mechanisms of this phenomenon is crucial for advancing stroke care. Pivotal to this discourse is the role of pericytes in modulating microvascular blood flow [5, 6, 7, 8]. The ESCAPE‐NA1 study highlighted the potential of preoperative NA‐1 administration to improve neurological prognosis in patients undergoing endovascular mechanical thrombectomy. This effect is particularly significant in those who do not receive intravenous thrombolysis [9]. NA‐1, acting as an inhibitor of the scaffold protein PSD95, decreases the threatening activation of nNOS, thus mitigating the excessive nitric oxide (NO) and peroxynitrite(ONOO^−^)production [10, 11]. Furthermore, ONOO^−^ may promote ET‐1 secretion through mechanisms such as oxidative nitrification stress and epigenetic regulation [12, 13, 14]. Meanwhile, pericytes express high levels of endothelin receptor A and contract robustly in response to ET‐1 [15, 16]. Notably, NA‐1 has been shown to exert protective effects not only on neurons but also on the blood–brain barrier (BBB) following ischemic injury [17]. Preservation of BBB integrity may synergistically mitigate neuroinflammation and secondary vascular dysfunction, potentially influencing pericyte‐mediated capillary constriction.

Given these insights, we hypothesized that NA‐1's interference with the PSD95 pathway could reduce microvessel contraction by pericytes through the suppression of endothelin secretion. Driven by this hypothesis and the pressing clinical need to mitigate the no‐reflow phenomenon post‐stroke, the present study was designed to explore and affirm the potential of NA‐1 in inhibiting pericyte‐driven vasoconstriction during and after cerebral ischemia–reperfusion events.

## 2 Materials and Methods

### 2.1 Ethics Statement

Beijing Neurosurgery Institute Laboratory Animal Welfare Ethics Committee approved the study design (approval NO. 2022201003) according to standards of animal welfare. We conducted our experimental procedures in strict compliance with laboratory animal welfare ethics laws and regulations, and we also improved these procedures to minimize harm to the animals.

### 2.2 Mice

Male BALB/c mice aged 8–12 weeks (25–30 g) were obtained from sibeifu (Beijing) Laboratory Animal Technology Co. Ltd. (Beijing, China). The animals were housed in an SPF‐grade experimental room. They had unrestricted access to food and water at room temperature (22°C ± 2°C) and were kept on a 12‐h light/dark cycle. We randomly divided the mice into four groups: sham, transient middle cerebral artery occlusion (tMCAO) for 1.5 h with or without NA‐1 administration, and tMCAO for 1.5 h with NA‐1 and ET‐1. Based on a previous study, tat‐NR2B9c/NA‐1 (GC30774, GlpBio) was administered at a dosage of 10 μMole/kg [18]. It was prepared in PBS at a concentration of 2 mM/mL and was intravenously administered via the tail vein in a volume of 5 μL/g at the beginning of reperfusion. ET‐1(HY‐P202, MCE) was administered at a 0.59 μg/Kg dose [14]. It was prepared in pure water at a concentration of 0.5 μg/mL and was intravenously administered via the tail vein after giving NA‐1. Mice designated for laser speckle contrast imaging (LSCI) underwent immunofluorescence staining after sacrifice, while those used for behavioral assessment were subjected to ELISA after sacrifice. The number of every group for each experiment was 4–6.

### 2.3 Mouse tMCAO Model

Anesthesia was induced with 3%–5% isoflurane. We adjusted the anesthetic concentration based on each animal's respiratory rate and pain reflexes. The tMCAO model used a 6–0 silicon rubber‐coated suture (602256PK5Re, Doccol) inserted into the internal carotid artery (ICA) to occlude the proximal middle cerebral artery (MCA) [19]. We believed this model was appropriate for studying recanalization and the physiological effects of large blood vessel occlusion in clinical patients. A longitudinal incision of about 1–1.5 cm was made to expose and separate the right common carotid artery (CCA), external carotid artery (ECA), and internal carotid artery (ICA).

The distal end of the external carotid artery (ECA) was ligated, while the proximal end remained open. The ECA ligation was cut, and the monofilament nylon suture was inserted into the ICA from the ECA stump. The suture was advanced into the middle cerebral artery (MCA) until mild resistance was felt, approximately 8–9 mm from the origin of the ECA within the ICA. After 1.5 h of ischemia, blood flow in the middle cerebral artery (MCA) was restored by withdrawing the monofilament, and the ECA stump was subsequently ligated. The same surgery was performed on the sham operation group without occlusion of MCA. Mice were placed on a heating pad to maintain a temperature of 37°C ± 0.5°C during the operation and recovery from anesthesia.

### 2.4 Laser Speckle Contrast Imaging (LSCI)

LSCI measurement was performed based on modality analysis of blurring effects with speckle patterns using a laser speckle blood flow imager (RFLSI III, Shenzhen Ruiward Life Technology CO. Ltd), as previously described [20]. Cortical perfusion was monitored before ischemia, during ischemia, and at 2 and 24 h after recanalization; the region of interest (ROI) was specifically chosen within the territory of the middle cerebral artery. Additionally, the relative cerebral blood flow (CBF) ratio of ipsilateral to contralateral was calculated using the formula: (ipsilateral CBF/contralateral CBF)/(baseline ipsilateral CBF/baseline contralateral CBF) for each perfusion measurement.

### 2.5 Tissue Preparation for Histological Imaging

At 24 h after recanalization, 100 μL ~70KD Rhodamine B isothiocyanate (RITC)‐dextran (R9379, sigma, 5% w/v) was injected via the tail vein 10 min before sacrifice. The brains were fixed overnight with 4% paraformaldehyde (PFA), followed by solutions containing 30% sucrose in 4% PFA for 48 h. The 50 μm‐thick coronal sections were collected using a Vibratome (CM1520, Leica). Additionally, we collected six 30 μm‐thick sections for other experiments.

### 2.6 Immunofluorescence Staining

Pericytes in free‐floating sections were labeled with a mouse aminopeptidase N/CD13 antibody (reconstituted at 0.2 mg/mL in sterile PBS, 1:50; R&D Systems AF2335). Additionally, the vascular basement membrane was labeled with an anti‐collagen IV antibody (1:250, Abcam, ab19808). MAP2 Rabbit mAb (1:200, cell signaling technology, 8707S) staining was used to assess the neurite density of the infarction area. Sections were incubated in sodium citrate antigen retrieval solution (Solarbio, C1032) at 95°C for 10 min for antigen retrieval, followed by blocking in 0.1 M PBS containing 5% donkey serum (Solarbio, SL050) and 0.3% Triton X‐100 for 1 h at room temperature. Then they were incubated with primary antibodies at 4°C overnight. After washing with PBS/Triton X‐100, the sections were incubated with secondary antibodies for 4 h at room temperature. The secondary antibodies were donkey anti‐goat Alexa Fluor 488 (1:500; Thermo Fisher Scientific, A‐11055), donkey anti‐rabbit Alexa Fluor 647 (1:500; Thermo Fisher Scientific, A‐31573), and donkey anti‐rabbit Alexa Fluor 488 (1:500; Thermo Fisher Scientific, A‐21206). After washing with PBS/Triton X‐100, the sections were mounted with an antifading mounting medium (Solarbio, S2100). Z‐stacks of pericytes and vascular analysis were obtained by laser confocal microscopy (LSM 710; Zeiss, Germany). Images of MAP2 staining were scanned by Vectra Polaris (PerkinElmer, Spokane, WA, United States) as a measurement of neuronal processes.

### 2.7 Cranial Window Surgery

For the effect of pericytes on microvascular contraction in vivo, A dental drill made a 3 mm diameter craniotomy above the somatosensory cortex (centered above the right somatosensory cortex −3 mm from Bregma, 3.5–4 mm lateral). The underlying dura was removed. Neuro Trace 500/525 dye, diluted to 1:10 in PBS (Thermo Fisher Scientific, N21480), was applied topically to the cortical surface for 10 min to label pericytes [21] The cranial window was sealed with round 3 mm glass coverslips glued onto a 4 mm glass coverslip. The remaining exposed skull surface was closed, and a custom‐made head plate was affixed to the skull using dental cement.

### 2.8 Two‐Photon Imaging

The cranial window was implanted before two‐photon imaging. Imaging was conducted right before, during ischemia, and at two and 24 h after recanalization using a two‐photon microscope (LSM 880, Axio Examiner; Zeiss). This microscope was equipped with a MaiTai two‐photon laser (S/N 20481, Spectra‐Physics) and a 20× water immersion objective (W Plan‐Apochromat 20×/1.0 DIC (UC) VIS‐IR M27 75 mm; Zeiss) and was controlled by ZEN 2011 software (black edition, Zeiss, Germany). During measurement, the mice were head‐fixed through the custom‐made head plate secured on a customized metal plate and kept under anesthesia as described above. 100 μL RITC‐dextran was injected intravenously in the tail vein 10 min before imaging. The observation was under excitation at 800 nm to visualize the vasculature. Z‐stacks of vessels were recorded at a resolution of 512 × 512 pixels with a frame scan time of 943.72 ms, covering a volume of approximately 425 × 425 × 120 μm^3^, with each z‐stack comprising 10 frames.

### 2.9 Image Analysis

The mean RITC‐dextran signal intensity was measured for each hemi section and re‐colored with pseudo color using ImageJ (1.53 t, National Institutes of Health, USA) software. This signal was assumed to assess brain tissue perfusion. Additionally, the ratio of ipsilateral to contralateral RITC‐dextran signal intensity was calculated. To evaluate pericyte involvement in the no‐reflow phenomenon, the capillary's length was measured from the point where the RITC‐dextran signal terminated to the neatest visible pericyte soma midway, so as to the probability distribution for each 5 μm bin of the capillary's length between the blockage and the closest pericytes and distance between adjacent pericytes [22]. Capillary diameters were measured at the blockage locations, pericyte soma site, and every 5 μm distance from pericyte soma (5, 10, 15, 20 μm) [23]. We plotted the microvessel diameter against the distance from the pericyte soma and assessed whether the slope of this plot significantly differed from zero, indicating whether the microvessel diameter changed significantly at various distances from the pericyte soma [22, 23]. Capillary diameter versus distance from pericyte soma in sham and tMCAO+vehicle groups were compared. The perfused vessels were analyzed by AgioTool 64 (version 0.6a) for perfused length and the ratio of perfused vessel volume. Starting from the bregma level, 30 μm thick frozen sections were collected continuously, and every seven brain slices were selected for the evaluation of the infarct size. The area of MAP2 negative, contralateral, and ipsilateral hemispheres were calculated using ImageJ (version 1.53 t, NIH, USA). ROIs were selected by the polygon selection tool manually. To account for brain edema, we calculated the edema‐adjusted infarction area using the formula: infarcted area × (contralateral hemisphere area/ipsilateral hemisphere area) [24]. The infarcted area ratio to the whole brain was obtained by infarct area/2 × contralateral hemisphere area [25].

### 2.10 Enzyme‐Linked Immunosorbent Assay (ELISA)

For ELISA analysis, brain tissue samples were harvested from the infarcted hemisphere 24 h after recanalization. Tissue samples were homogenized in ice‐cold lysis buffer with a protease inhibitor cocktail (P8340, Sigma). Homogenates were centrifuged at 12,000 *g* for 20 min at 4°C, and the supernatant was retained for ELISA analysis. Protein concentration was measured by dicinchoninic acid assay (23,227, Thermo). ET‐1 and ONOO^−^ levels were quantified using commercial ELISA kits (E‐EL‐M2730, Elabscience for ET‐1 and K6‐03883, KIRBIO for ONOO‐) according to the manufacturer's protocol. Briefly: Standards and samples were added to pre‐coated 96‐well plates in triplicate and incubated according to the kit protocol. After washing with detergents 100 μL/well of biotinylated detection antibody was added and incubated for 1 h at 37°C. After washing plates were incubated with streptavidin‐HRP conjugate (30 min, 37°C), and developed with TMB substrate (15 min, dark). Reactions were stopped with stop solution, and absorbance was measured at 450 nm using a microplate reader. Target protein concentrations (pg/mL for ET‐1 and ng/mL for ONOO^−^) were calculated from the standard curve and normalized to total protein content (pg/mg protein for ET‐1 and ng/mL protein for ONOO^−^). Statistical significance was assessed using one‐way ANOVA with post hoc Tukey's test (GraphPad Prism v9.4.0).

### 2.11 Behavioral Assessment

The behavioral test was performed 24 h after recanalization using a modified neurological severity score (mNSS). The mNSS was graded on a scale of 0 to 18 (with a higher score representing more severe injury). In this scoring system, a score of 0 indicates normal function, 1–6 indicates mild injury, 7–12 indicates moderate injury, and 13–18 indicates severe injury. It reflected various aspects of neurologic function, including motor, sensory, balance, and reflex tests [26, 27]. The information has been summarized and included in Table 1.

**TABLE 1:** Modified neurological severity score.

Exercise test	
Tail lift experiment	
Forelimb flexion	1
Hindlimb flexion	1
Head deviates > 10° from vertical axis within 30s	1
Place the mouse on the ground	
Walk normally	0
Can't walk in a straight line	1
Circle to the paralyzed side	2
Falling to the paralyzed side	3
Sensory test	
Placement test (visual and tactile test) (10 cm away from the tabletop at a 45° tilt close to the tabletop, response delay is positive)	1
Proprioception test (deep sensation, pulling the paw toward the edge of the table to stimulate limb muscles)	1
Balance Beam Trial (Normal 0, Max 6)	
Stable balance posture	0
Hold on to the edge of the balance beam	1
Hold on to the balance beam, one limb hangs down	2
Hold the balance beam tightly, hang down with two limbs or rotate on the balance beam (> 60s)	3
Tries to balance on a balance beam but falls (> 40s)	4
Tried to balance on a balance beam but fell (> 20s)	5
Falling, not attempting to balance on beam (< 20s)	6
Loss of reflexes and abnormal movements	
Pinna reflex (shaking head when touching the external auditory canal)	1
Corneal reflex (blinking when the cotton wool touches the cornea)	1
Startle reflex (motor response to snapping cardboard noises)	1
Seizures, myoclonus, dystonia	1
Maximum score	18

*Note:* The modified neurological severity score (mNSS) was selected to serve as a methodology for assessing neurological deficits with balance, movement, reflex, or sensation problems in rodent models.

### 2.12 Statistical Analysis

Statistical analysis was performed using GraphPad Prism (version 9.4.0). Continuous variables were compared using independent samples *t*‐test (for two‐group comparisons) or one‐way ANOVA (multi‐group comparisons with one independent variable factor) or two‐way ANOVA (multi‐group comparisons with two independent variable factor) after confirming normality (Shapiro–Wilk test) and homogeneity of variance (Levene's test). Nonparametric alternatives, such as the Mann–Whitney U test or the Kruskal–Wallis test, were used when the assumptions of normality or homogeneity were not met. All statistical tests and the group size (*n*) were listed in the figure legends. The mean ± standard error of the mean (SEM) was used to express the results in bar plots and graphs. *p* < 0.05 was considered statistically significant.

## 3 Results

### 3.1 NA‐1 Improves Blood Flow After Ischemia and Recanalization

**FIGURE 1:**
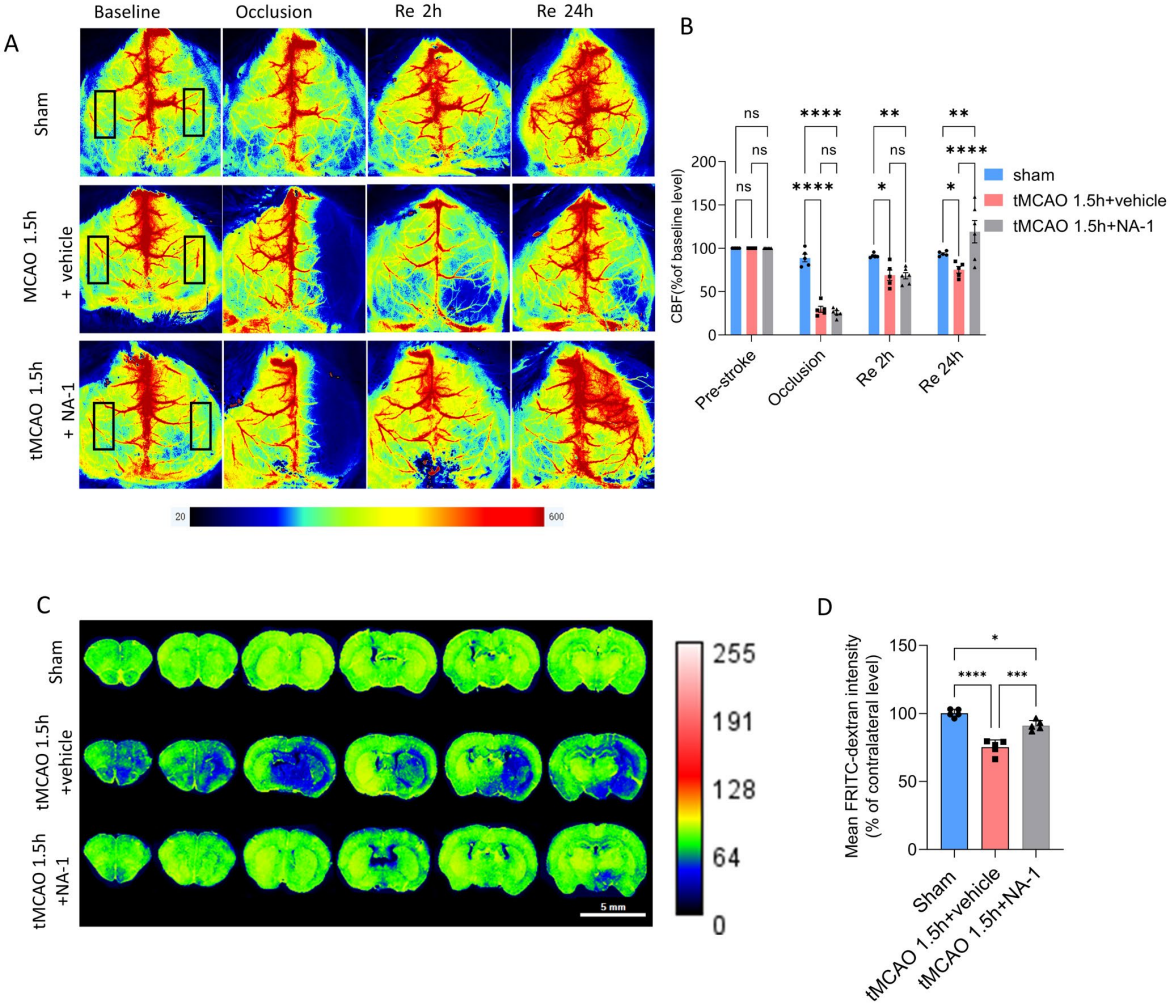
NA‐1 improves perfusion in the ischemic brain. (A) Representative LSCI images show mouse cortical perfusion in the sham, tMCAO 1.5 h + vehicle, and tMCAO 1.5 h + NA‐1 groups at various time points. (B) Average ROI from (A) compared with baseline levels. *N* = 5–6/group. Shown in mean ± SEM. *vehicle group versus NA‐1 group, ****p < 0.0001, two‐way ANOVA. (C) Low power view of mouse brain sections with an experimental infusion with RITC‐dextran (re‐colored with pseudo color) into sham, tMCAO + vehicle, or tMCAO + NA‐1 group. (D) The ratio of mean RITC‐dextran intensity in the ipsilateral versus contralateral hemisphere brain slices from the sham, tMCAO + vehicle, and tMCAO + NA‐1 groups was used to assess cerebral perfusion. *N* = 5/group. Shown in mean ± SEM. *p < 0.05, ***p < 0.001, ****p < 0.0001, one‐way ANOVA.

LSCI indicated cortical perfusion decreased by ~70% during ischemia in both tMCAO+vehicle and tMCAO+NA‐1 groups (Figure 1A,B). Twenty‐four hours after recanalization, blood flow recovered to ~75.3% of the baseline level for the tMCAO 1.5 h plus vehicle group and ~ 122.5% of the baseline level for the tMCAO 1.5 h plus NA‐1 group (tMCAO 1.5 h plus NA‐1 group significantly increased).

**FIGURE 2:**
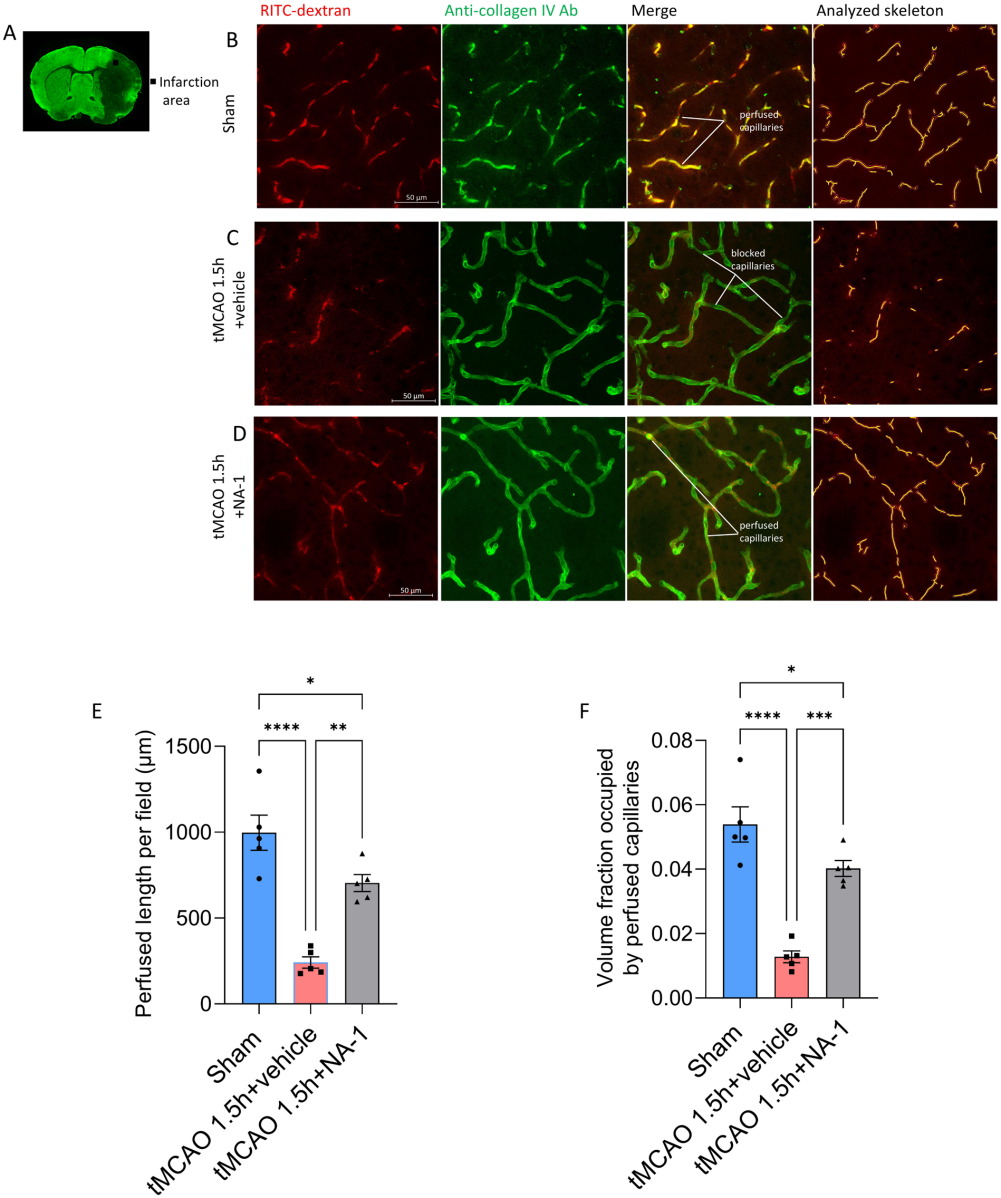
NA‐1 improves neural and microvascular integrity. (A) MAP‐2 immunofluorescence staining indicated neurite density loss of infarction area (MAP‐2 negative). (B–D) Representative images of the microcirculation in the infarction area as in (A) groups of sham (B), tMCAO+vehicle and 24 h recanalization (C), and tMCAO+NA‐1 and 24 h recanalization (D). Images showing RITC‐dextran labeling (red) within vessels under perfusion, anti‐collagen IV antibody (green) labeling positions of all vessels, a merge of the RITC‐dextran and anti‐collagen IV antibody, and the skeleton analysis (indicated with yellow) of any perfused microvessel. Scale bar = 50 μm. (E, F) 24 h after recanalization, the total perfused capillary length (E) and the overall volume fraction occupied by perfused capillaries (F) in 50 μm deep confocal Z‐stacks compared within the sham group (19 stacks), tMCAO+vehicle group (27 stacks) and tMCAO+NA‐1 group (23 stacks). Five animals in each group. Shown in mean ± SEM. *p < 0.05, **p < 0.01, ***p < 0.01, ****p < 0.0001, one‐way ANOVA.

Twenty‐four hours after recanalization, the volume of perfused vessels in fixed brain slices was evaluated by comparing the ipsilateral and contralateral RITC‐dextran signal intensity. This analysis revealed that brain ischemia and the recanalization process resulted in no‐reflow, with the perfusion blood volume decreasing by approximately 25% (*p* < 0.0001 compared to the sham group, Figure 1C,D). NA‐1 significantly inhibited the decrease of ipsilateral perfusion (Figure 1C,D), but this effect was counteracted notably by ET‐1 administration (Figure S1A,B). Higher magnification imaging showed that the drop in blood volume after ischemia and recanalization was connected to a significant reduction in capillary perfusion (Figure 2). The total perfused capillary length in a 50 μm deep confocal Z‐stack (frame size 212.45 × 212.45 μm) was reduced to 24% (sham 997 ± 103 μm versus tMCAO 1.5 h + vehicle 241 ± 33 μm, *p* = 0.0001), and the overall volume fraction occupied by perfused capillaries was reduced to ~24% (sham 0.0539 ± 0.0055 versus tMCAO 1.5 h + vehicle 0.0128 ± 0.0018, *p* < 0.0001, Figure 2E,F). Disruption of PSD95 during reperfusion led to an increase in both the length of perfused capillaries and the volume fraction they occupied. Specifically, the length of the perfused capillaries was 704 ± 49 μm, and the volume fraction was 0.0402 ± 0.0025 (Figure 2).

### 3.2 Pericytes Constrict Capillaries After Ischemia and Recanalization

**FIGURE 3:**
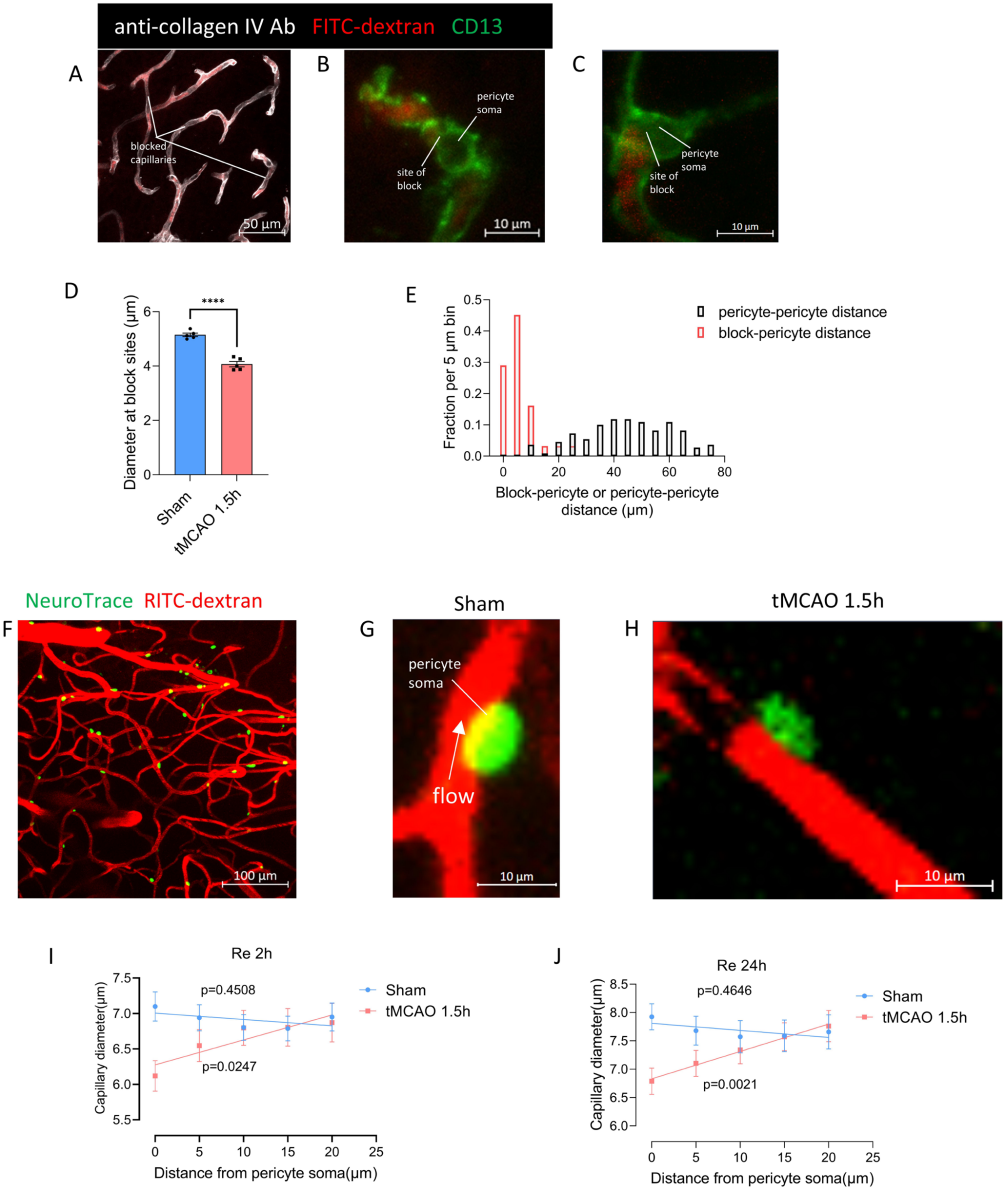
Pericytes constrict capillaries after ischemic recanalization. (A) Capillaries in slices of the BALB/c mouse brain were perfused with RITC‐dextran (red) and labeled with an anti‐collagen IV antibody (white); the RITC‐dextran labeling indicated perfused vessels; White lines indicated blocked vessels. Scale bar = 50 μm. (B, C) Representative images of capillary blockage near pericyte soma. Pericytes are labeled by antibody to CD13 (green). Scale bar = 10 μm. (D) Diameter at block sites. The number of the block was 41 and 42 in the sham and tMCAO 1.5 h groups respectively. Shown in mean ± SEM. **** *p* < 0.0001, student's *t*‐test. (E) The probability distribution was calculated for each 5 μm bin, measuring the distance between the obstruction and the nearest pericyte soma 24 h after recanalization(data from 31 blocked sites). Additionally, the separation between nearby pericytes was analyzed (data from 110 pericyte pairs). (F) Representative two‐photon in vivo imaging stack of the BALB/c mouse cortex vessels, showing pericytes labeled by NeuroTrace 500/525 (green) and flow labeled by intravenous RITC‐dextran (red). Scale bar = 100 μm. (G, H) Higher magnification images of the pericyte within the cortical capillaries in sham or 1.5 h ischemia (recanalization for 2 h). The data could indicate a capillary block near pericyte soma. Scale bar = 10 μm. (I, J) Capillary diameter versus total distance from the pericyte soma. The number of pericytes counted slides was 22 and 25 (I) and 23 and 22 (J) respectively for ischemia and sham, 10 stacks each. Shown in mean ± SEM.

At higher magnification, images revealed that while some capillaries were fully perfused within the assessed area, others were completely unperfused. Furthermore, some capillaries abruptly ceased blood flow, which was accompanied by a decrease in RITC‐dextran intensity over several microns(Figure 3A–C). At block sites, the diameter of the anti‐collagen IV antibody labeling lumen at the ultimate position blood reached was considerably lower in tMCAO 1.5 h capillaries compared with that in the sham group (sham 5.15 ± 0.15 μm versus tMCAO 1.5 h 4.07 ± 0.22 μm; *p* < 0.0001, Figure 3D). Thus, blockage developed due to capillary constriction brought on by ischemia; it endures even after recanalization.

Aminopeptidase N/CD13 antibody labeling indicated that many capillary blockages were near the pericyte somas (Figure 3B,C). The distance of 31 jams to the nearest pericyte soma was then measured. This distance's distribution was compared to the inter‐pericyte distribution in Figure 3E (if blocks failed to rely upon pericytes, the likelihood distribution of the blockage‐pericyte would be constant till half the distance between pericytes). The mean blockage–pericyte length was 5.62 μm after ischemia and recanalization, a smaller amount than a quarter of the distance between pericytes (44.81 μm). These data suggested that pericytes constricted capillaries led to the capillary block.

In the sham group, the mean distance from blockage to the neatest pericyte soma was more extensive (14.1 ± 5.3 μm, *p* < 0.0001 compared to tMCAO 1.5 h and recanalization), indicating a different block mechanism in the sham condition.

**FIGURE 4:**
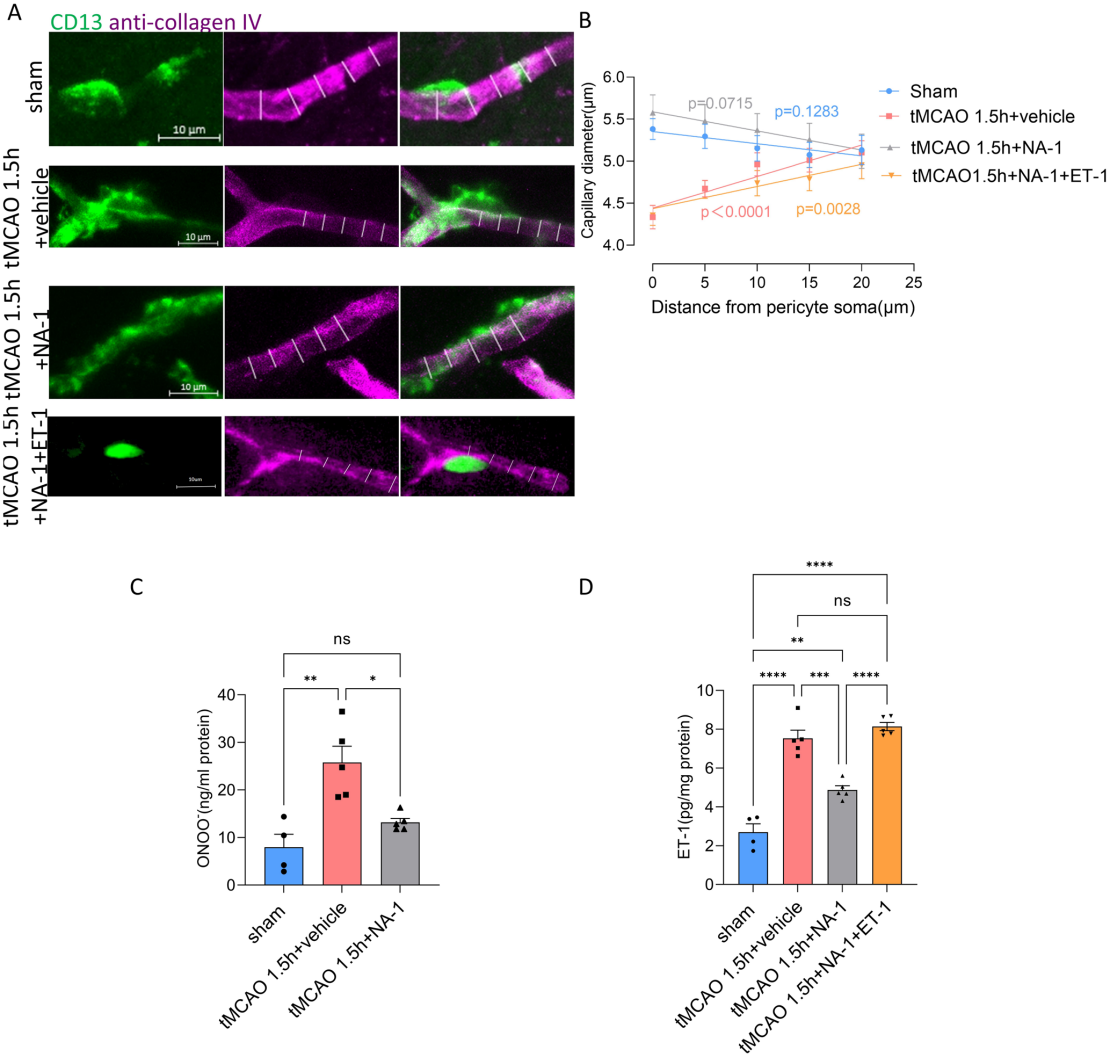
NA‐1 decreases ET‐1‐mediated pericytic vasoconstrictions after ischemic recanalization. (A) Images showing capillaries at the pericyte soma for the sham, tMCAO + vehicle, and tMCAO + NA‐1 groups. Pericytic components were labeled with CD13 (green), and anti‐collagen IV antibody (purple) labeled vessels. White lines indicated capillary diameter. (B): The relationship between capillary diameter and the distance from a visible pericyte soma in the sham, tMCAO + vehicle, tMCAO + NA‐1, and tMCAO + NA‐1 + ET‐1 groups is shown (with 26, 25, 25, and 13 pericytes counted, respectively). Shown in mean ± SEM. (C, D) ELISA results and quantitative analysis on ONOO^−^ and ET‐1 expression of sham, tMCAO + vehicle, tMCAO + NA‐1, and tMCAO + ET‐1 groups. Four to five animals in each group. Shown in mean ± SEM. **p < 0.01, ***p < 0.01, ****p < 0.0001, one‐way ANOVA.

To assess pericyte‐mediated capillary constriction further, the anti‐collagen IV antibody labeling lumen diameter at 5 μm intervals from pericyte soma was measured [22, 23]. After ischemia for 1.5 h and recanalization, The diameter was considerably shrunk (by 19%, *p* < 0.0001) at the pericyte soma site compared with controls but less reduced further from the soma. After ischemia and recanalization, the diameter significantly increased with distance from the soma (*p* < 0.0001 when comparing the slope of the best‐fit tMCAO 1.5 h regression line to zero). However, this increase was not observed in the sham group (*p* = 0.1283; Figure 4B). This suggests that pericytes are responsible for the diameter reduction, indicating that constriction occurs primarily near the pericyte soma. Such constriction increases vascular resistance, directly reducing blood flow. Additionally, it may lead to the accumulation of blood cells in the narrower lumen, which could occlude the vessel and further diminish blood flow [22, 23].

### 3.3 Pericytes Constrict Brain Cortical Capillaries After Ischemia and Recanalization

Using two‐photon in vivo imaging with Neuro Trace 500/525‐labeled mouse pericytes, we observed cortical pericytes constricting and obstructing capillaries after ischemia and recanalization (Figure 3C) [21]. The distance of 16 jams to the nearest pericyte soma was measured to quantify whether the blockages occurred disproportionately close to pericytes. This distance was 5.8 ± 6.7 μm, which is only 10% of the mean distance between brain cortical pericytes (58.7 ± 22.4 μm). The plot of capillary diameter against distance from the pericyte soma (Figure 3I,J) revealed that 2 h after tMCAO 1.5 h and recanalization reduced the diameter at the soma by 14% (sham: 7.1 ± 1.0 μm; tMCAO: 6.1 ± 1.0 μm, *p* = 0.002). In contrast, there was no significant change in diameter 10 μm away from the soma (sham: 7.0 ± 1.0 μm; tMCAO: 6.9 ± 1.3 μm, *p* = 0.8097). When the time came to 24 h after recanalization, the diameter reduced by 14% at pericyte soma (sham 7.9 ± 1.1 versus tMCAO 1.5 h and recanalization 24 h 6.8 ± 1.1 μm, *p* = 0.0012) with no significant effect on the diameter far from the soma (sham 7.7 ± 1.4 μm versus tMCAO 1.5 h and recanalization 24 h 7.8 ± 1.3 μm, *p* = 0.8040). The diameter increased considerably with distance from the pericyte soma after tMCAO 1.5 h and recanalization 2 h (*p* = 0.0247 contrasting the best‐fit regression line's slope with zero). In contrast, in the sham group, diameter decreased insignificantly with distance (*p* = 0.4508). The result at the time of recanalization 24 h was similar (*p* = 0.0021 contrasting the best‐fit regression line's slope with zero), while in the sham group, diameter decreased insignificantly with distance (*p* = 0.4646). The data supported capillaries constricted specifically near cortical pericytes.

### 3.4 NA‐1 Reduces Pericyte‐Mediated Vasoconstriction and Endothelin‐1 After Ischemia and Recanalization

To assess the effect of NA‐1 on pericyte constriction in capillaries, we measured capillary diameters labeled with anti‐collagen‐IV antibodies. Measurements were taken at 5‐μm intervals from the pericyte soma in the NA‐1‐treated tMCAO group. These results were then compared to those from the sham, tMCAO + vehicle, and tMCAO + NA‐1 + ET‐1 groups (Figure 4A,B). The tMCAO + NA‐1 group exhibited a trend similar to the sham group, with capillary diameter not significantly decreasing as the distance from the pericyte soma increased (slope of the best‐fit regression line compared with zero, *p* = 0.0715). This effect was counteracted notably by ET‐1 administration (Figure 4B). It was suggested that NA‐1 decreases ET‐1‐mediated pericytic vasoconstrictions after ischemic recanalization.

To further elucidate the mechanism of NA‐1 inhibition of pericyte‐mediated vasoconstriction after ischemia and recanalization, ONOO^−^ and endothelin‐1 were analyzed by ELISA. Endothelin‐1 was a vasoconstrictor peptide mainly secreted by endothelial cells, which acted on ETA receptors on pericytes, causing strong contraction of pericytes [28]. The levels of ONOO^−^ and endothelin‐1 were significantly lower in the tMCAO+NA‐1 group than in the tMCAO 1.5 h + vehicle group. Specifically, ONOO^−^ levels were 13.1871 ± 0.8337 ng/mL in the tMCAO+NA‐1 group versus 25.7928 ± 3.4184 ng/mL in the MCAO + vehicle group (*p* = 0.0012). Endothelin‐1 levels were 4.8679 ± 0.2175 pg/mL in the tMCAO+NA‐1 group compared to 7.5300 ± 0.4223 pg/mL in the MCAO + vehicle group (*p* = 0.0002) (Figure 4C,D). However, when added ET‐1, this effect of NA‐1 was notably counteracted After NA‐1 administration, the synthesis of ONOO^−^ and endothelin‐1 decreased, which inhibited pericyte‐mediated vasoconstriction following ischemia and recanalization.

### 3.5 NA‐1 Reduces Infarction Volume and Behavior Defects

**FIGURE 5:**
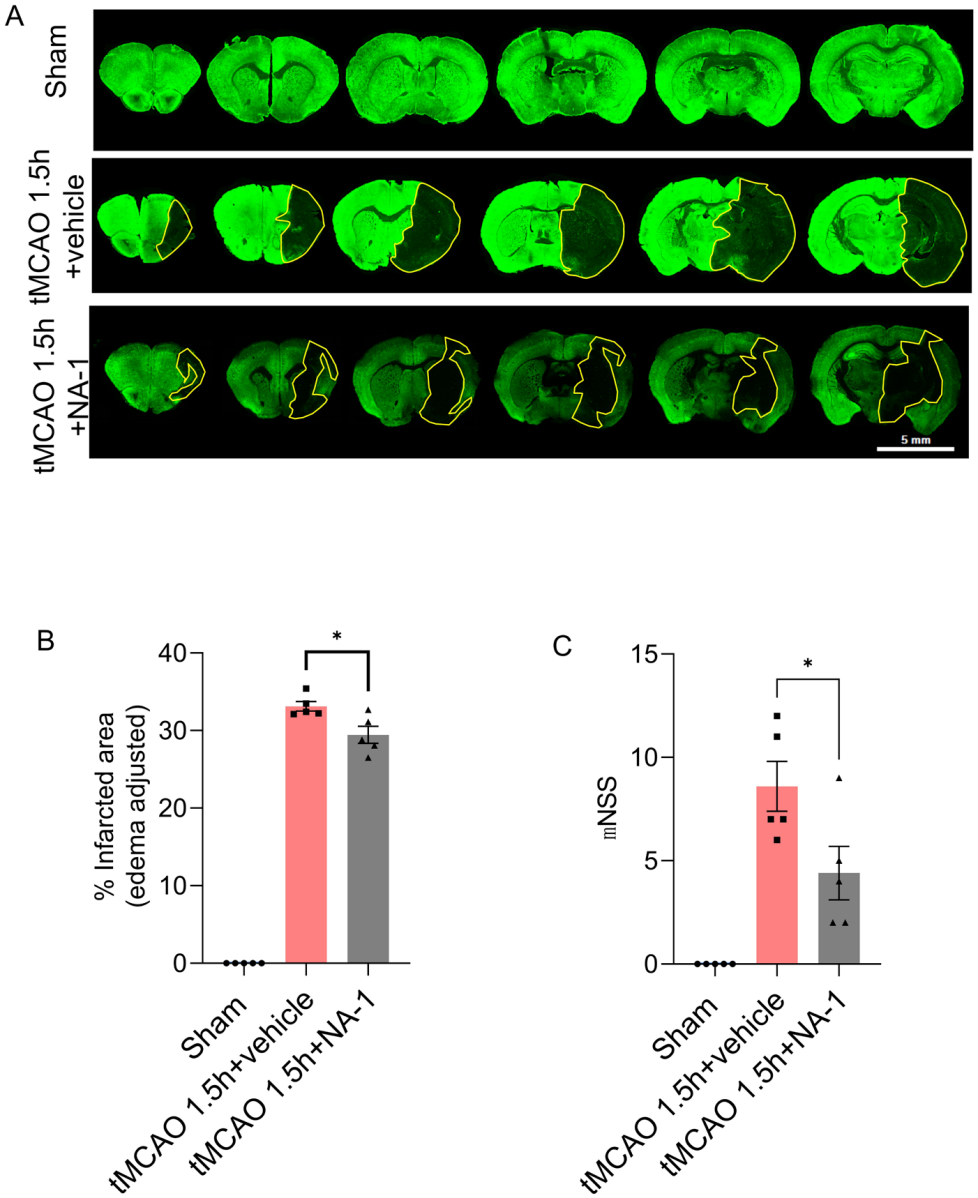
NA‐1 reduces infarction area and behavior deficits. Neuronal processes were utilized in the measurement during the injury and neurovascular repair. (A) MAP‐2 immunofluorescence staining indicated neurite loss of the infarction area (MAP‐2 negative with yellow coil painting). (B) Calculation and demonstration (%) of the infarcted area from the sham, tMCAO+vehicle, or tMCAO+NA‐1 group at 24 h after recanalization. *N* = 5–6/group. Shown in mean ± SEM. *p < 0.05, student's *t*‐test. C: MNSS assessment in sham, tMCAO+vehicle, or tMCAO+NA‐1 group at 24 h after recanalization. *N* = 5‐6/group. Shown in mean ± SEM. *p < 0.05, student's *t*‐test.

To further evaluate the prognostic impact of NA‐1 administration, MAP2 staining was used to calculate the infarct volume after ischemia and recanalization at 24 h. The infarct volume was significantly reduced in the tMCAO 1.5 h plus NA‐1 group as compared with the control group (*p* = 0.0083) (Figure 5A,B).

Behavioral outcomes were assessed using the mNSS score 24 h after vascular recanalization. The tMCAO without treatment group exhibited moderate behavioral deficits with an average score of nine. In contrast, the tMCAO+NA‐1 group displayed milder behavioral deficits, recording an average score of four. Consequently, ischemic animals treated with NA‐1 showed a significant improvement in behavioral outcomes as compared to those in the vehicle‐treated group (*p* = 0.0213) (Figure 5C).

## 4 Discussion

Our study demonstrates that after cerebral ischemia‐recanalization, pericyte‐mediated vasoconstriction leads to capillary stenosis‐associated microcirculation impairment, which results in the no‐reflow phenomenon. Administration of NA‐1 treatment, however, inhibits pericyte‐mediated vasoconstriction after ischemia–reperfusion by reducing endothelin‐1 synthesis, thereby improving no‐reflow after vascular opening, increasing cerebral perfusion, reducing infarct size, and decreasing behavioral deficits in mice.

There is controversy about whether pericytes are associated with capillary contraction [8, 29, 30, 31]. Recent studies have clarified the contractility and kinetics of the two main pericyte subtypes [32, 33]. They have also observed the contraction of specialized capillary pericytes following ischemia–recanalization [34, 35]. Capillaries after ischemia–recanalization have also been found in studies in the heart and kidney [22, 36]. While a study finds capillary diameter at the site of the pericyte soma can remain in the penumbra zone after ischemia–recanalization [30]. These controversies are consistent with the lack of methods to specifically identify pericytes, different naming of pericyte subtypes, differences in the site of observation, and the difficulties in identifying small changes in capillary diameter. Our study uses ROIs for the penumbra zone developed into an infarcted area 24 h after ischemia.

NA‐1 is an inhibitor of PSD95. Previous studies have focused on the neuroprotective effects of NA‐1, confirming that NA‐1 is an effective neuroprotective agent [37]. ENACT is a phase II clinical study that confirms the neuroprotective effects of NA‐1 in ischemic stroke in patients with aneurysms [38]. A subsequent phase III clinical trial, ESCAPE‐NA‐1, has suggested a neuroprotective effect of NA‐1 in patients with mechanical embolization [9]. Our study reveals that NA‐1 may indirectly inhibit pericyte‐mediated vasoconstriction and improve the no‐reflow phenomenon following ischemia–recanalization. This finding offers new insights for future clinical studies on NA‐1, complements its mechanism for improving cerebral ischemia prognosis, and supports its potential clinical application.

There are some vasoactive mediators known to act on pericytes. ATP may act directly on purinergic receptors expressed by pericytes and induce pericyte‐mediated vasoconstriction [31, 39]. Noradrenaline may act on other cell types like astrocytes, which secondarily signal pericytes to constrict [6]. Signaling pathways initiated by arachidonic acid metabolism, such as the production of prostaglandin H2 and thromboxane A2, can potently contract capillary pericytes [40, 41]. Endothelin receptor A is abundant in pericytes, which causes contraction in response to endothelin‐1 (endothelin‐1) [28]. Glutamate activates arachidonic acid metabolism in astrocytes or neurons to produce prostaglandin E2, activating prostaglandin E2 receptor 4 on pericytes and inducing nitric oxide production to promote vasodilation [40, 42]. Adenosine may also cause the relaxation of pericytes by promoting KATP potassium channel flux via A2a purine receptors [43]. Furthermore, our study indicates that NA‐1 reduces pericytic vasoconstriction induced by endothelin‐1 during the process of ischemic recanalization.

In vitro primary rat brain pericytes [44] and isolated rat retinal microvascular pericytes [45], as well as in vitro human microvascular pericytes in response to ROS [46], ischemic rat cerebellar slices [47], and pericytes from mice after ischemic stroke [7] show an increase in intracellular calcium ions in response to voltage‐gated calcium channels. Increased intracellular calcium ions in pericytes can promote contraction, with downstream signals through calmodulin and MLC kinase to phosphorylate MLC and induce contraction, similar to the signaling pathway in smooth muscle cells of isolated rat cerebral arteries. Single‐cell ribonucleic acid sequencing analysis of mouse cortex has shown that Myl9 (encoding MLC regulatory polypeptide 9) is expressed in pericytes [48]. Treating isolated rat retinal microvascular pericytes with cyclic guanosine monophosphate (cGMP) analogs can inhibit voltage‐gated calcium channels and calcium‐gated chloride channels. This treatment reduces intracellular calcium levels and whole‐cell calcium and chloride currents, promoting pericyte relaxation [45]. Our study indicates that the synthesis of endothelin‐1 decreases following the administration of NA‐1. This effect may occur by reducing the binding of endothelin‐1 to its receptors on pericytes, which in turn decreases calcium influx, inhibits downstream signaling by calmodulin and MLC kinase, and ultimately reduces the phosphorylation of MLC, leading to decreased contraction.

## 5 Conclusion

Our study presents initial evidence that NA‐1 treatment can decrease endothelin‐1 levels and counteract pericyte‐driven vasoconstriction. This action enhances cerebral perfusion and addresses the no‐reflow phenomenon post‐ischemia‐recanalization, ultimately diminishing infarct size and behavioral deficits. This sheds light on a novel mechanism through which NA‐1 can enhance the prognosis of ischemic stroke. This study lays the groundwork for future clinical trials on NA‐1 and offers new insights into alleviating the no‐reflow phenomenon following ischemia‐recanalization.

## Author Contributions

X.Y. and L.L. conceived and designed the study. X.Y., H.T., and J.Z. performed all the experiments and analyzed the data. L.L. supervised the work. X.Y. drafted the manuscript; L.Z., X.L., and J.Z. reviewed the manuscript. Z.Z.W., Y.D., and L.L. edited the manuscript. L.L. and X.N. provided financial support.

## Ethics Statement

Beijing Neurosurgery Institute Laboratory Animal Welfare Ethics Committee approved the study design (approval NO. 2022201003) according to standards of animal welfare.

## Conflicts of Interest

The authors declare no conflicts of interest.

## Supporting information


**Figure S1.** ET‐1 interferes with NA‐1’s function in promoting blood flow in the ischemic brain.

## Data Availability

The authors declare that all other data supporting the results of this study are available in the article, in the online Supporting Information, and from the authors upon request.
