# Supplementary material for: Impact of NA‐1 on Pericyte‐Driven Vasoconstriction and Its Role in No‐Reflow During Cerebral Ischemia–Reperfusion
Source: CNS Neurosci Ther. 2025 May 25;31(5):e70409. doi: 10.1111/cns.70409 (PMC12104562; doi:10.1111/cns.70409)
Supplement: Supplementary file 1 — Figure S1. ET‐1 interferes with NA‐1’s function in promoting blood flow in the ischemic brain. [file CNS-31-e70409-s001.docx]

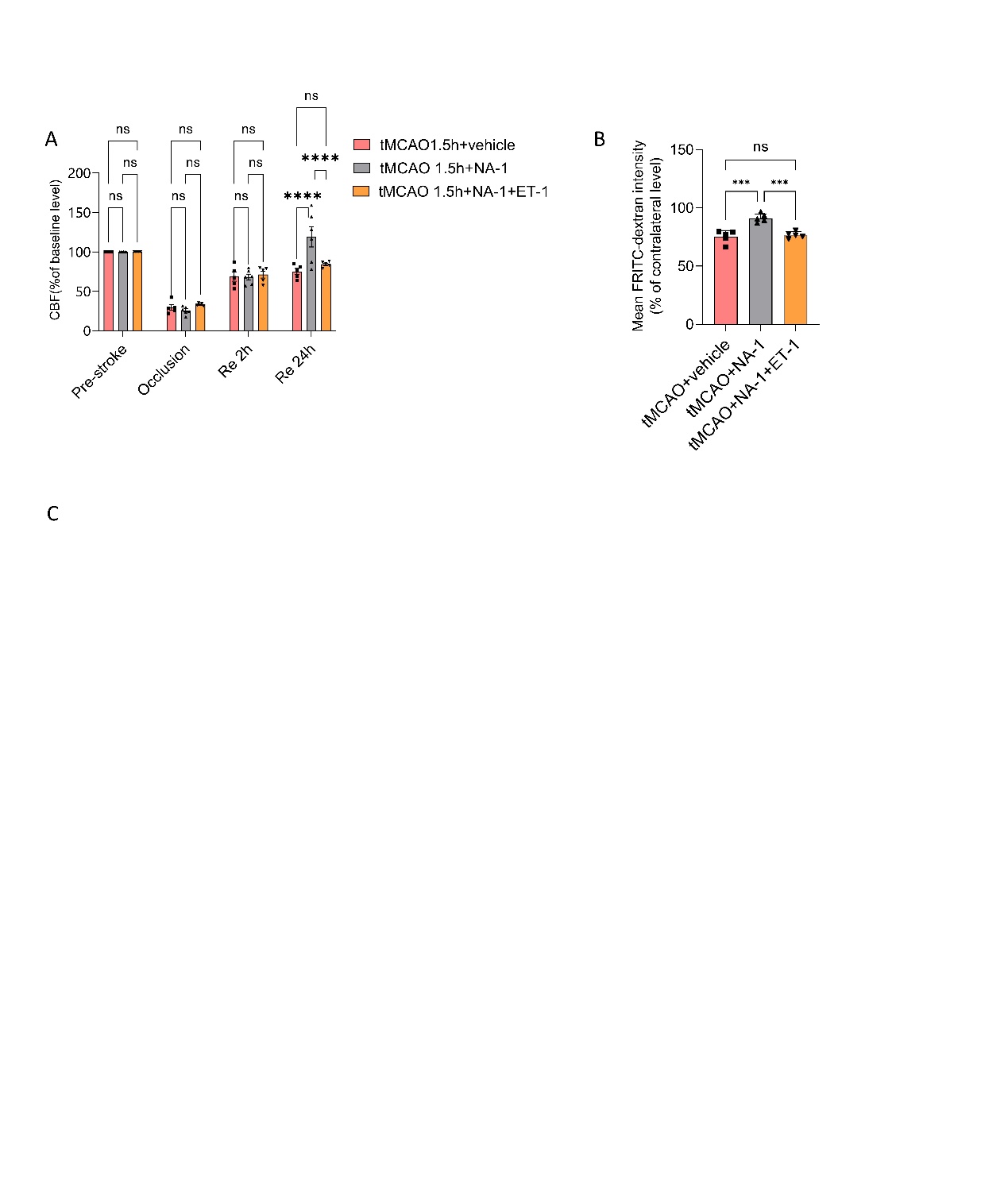


**Figure S1: ET-1 interferes with NA-1's function in promoting blood flow in the ischemic brain.**

A: LSCI images indicated mouse cortical perfusion from tMCAO 1.5h + vehicle, tMCAO 1.5h + NA-1, and tMCAO 1.5h + NA-1 +ET-1 group with a different time point after stroke. N = 5/group. Shown in mean ± SEM. ****p＜0.0001, two-way ANOVA. B: The ratio of mean RITC-dextran intensity in ipsilateral/contralateral hemisphere brain slices from tMCAO 1.5h + vehicle, tMCAO 1.5h + NA-1 and tMCAO 1.5h + NA-1 +ET-1 group served as a measure of cerebral perfusion. N = 5/group. Shown in mean ± SEM. ***p<0.001, one-way ANOVA.
